# Effect of providing purple sweet potato water extract on tumor necrosis factor-α levels, protein 53 expression, glial fibrillary acidic protein expression, brain-derived neurotrophic factor levels, and spatial working memory in rats with d-galactose induction

**DOI:** 10.1590/1980-5764-DN-2021-0090

**Published:** 2022-06-06

**Authors:** Ketut Widyastuti, Tjokorda Gde Bagus Mahadewa, Dewa Ngurah Suprapta, Anak Agung Raka Sudewi

**Affiliations:** 1 Udayana University, Medical Faculty, Department of Neurology, Bali, Indonesia.; 2 Udayana University, Medical Faculty, Department of Neurosurgery, Bali, Indonesia.; 3 Udayana University, Faculty of Agricultural, Laboratory of Biopesticide, Bali, Indonesia.

**Keywords:** Galactose, Tumor Necrosis Factor-alpha, Genes, p53, Glial Fibrillary Acidic Protein, Brain-Derived Neurotrophic Factor, Spatial Memory, Galactose, Fator de Necrose Tumoral alfa, Genes p53, Proteína Glial Fibrilar Ácida, Fator Neurotrófico Derivado do Encéfalo, Memória Espacial

## Abstract

Alzheimer's dementia (AD) is a neurodegenerative disease. The mechanism of oxidative stress in AD is due to amyloid beta (Aβ) protein that aggregates to form plaques, which further triggers chronic inflammation and neuronal apoptosis. Purple sweet potato extract with the main content of anthocyanins is a potential antioxidant with a direct target on the amyloid cascade hypothesis.

**Objective:**

The research objective was to determine the role of purple sweet potato water extract as an antioxidant and anti-inflammatory in preventing apoptosis in order to provide a neuroprotective effect in d-galactose-induced rats.

**Methods:**

A total of 100 male Wistar rats with randomized posttest-only control group design that met the eligibility criteria were included in this study. The treatment group was given 200 mg/kg BW/day of purple sweet potato water extract on days 1–70. d-galactose induction was administered in the treatment and control groups on days 15–70.

**Results:**

The independent t-test showed that the mean tumor necrosis factor-α (TNF-α) levels in the treatment group (735.36±139.74) was significantly lower than that in the control group (896.77±152.52). The p53 and glial fibrillary acidic protein (GFAP) expressions of astrocyte cells in the treatment group were significantly lower than that in the control group. The brain-derived neurotrophic factor (BDNF) levels in the treatment group (498.13±121.47) were higher than that in the control (391.93±140.28), and there was a significant increase in spatial working memory in the treatment group (72.01±10.22) than the control (59.77±11.87).

**Conclusions:**

The neuroprotective effect of purple sweet potato extract is due to d-galactose induction resulting from decrease in TNF-α levels, p53 expression, and GFAP expression and increase in BDNF levels and spatial working memory.

## RESUMO.

A doença de Alzheimer (DA) é uma doença neurodegenerativa. O mecanismo de estresse oxidativo na DA ocorre devido à proteína beta amilóide que se agrega para formar placas que desencadeiam inflamação crônica e apoptose neuronal. O extrato de batata-doce roxa composto principalmente por antocianinas é um potencial antioxidante com efeito direto sobre a hipótese da cascata amilóide.

**Objetivo:**

O objetivo da pesquisa foi determinar o papel do extrato aquoso de batata-doce roxa como antioxidante e anti-inflamatório na prevenção da apoptose, para proporcionar um efeito neuroprotetor em ratos induzidos por D-galactose.

**Métodos:**

Grupo controle randomizado pós-teste com 100 ratos Wistar machos que preencheram os critérios de elegibilidade. O grupo de tratamento recebeu 200mg/kg de peso corporal/dia de extrato aquoso de batata-doce roxa nos dias 1-70. A indução de D-galactose foi testada nos grupos de tratamento e controle nos dias 15-70.

**Resultados:**

O teste t independente mostrou que a média dos níveis de TNF-α no grupo de tratamento (735,36±139,74) foi significativamente menor do que no grupo controle (896,77±152,52). A expressão de p53 e a expressão de GFAP de células de astrócitos foram significativamente menores no grupo de tratamento do que no grupo controle. Os níveis de BDNF no grupo de tratamento (498,13±121,47) foram maiores que no grupo controle (391,93±140,28) e houve um aumento significativo da memória de trabalho espacial no grupo de tratamento (72,01±10,22) em relação ao controle (59,77±11,87).

**Conclusões:**

O efeito neuroprotetor do extrato de batata-doce roxa é devido à indução de D-galactose pela diminuição dos níveis de TNF-α, expressão de p53 e expressão de GFAP, aumentando assim os níveis de BDNF e memória espacial.

## INTRODUCTION

Increasing life expectancy causes an increase in the number of elderly people and problems related to the aging process. Dementia is a neurodegenerative disease that causes cognitive and behavioral disorders, affecting the elderly in their social and work activities. People with dementia experience dependence on their daily activities and become a burden to their family, community, and government. Efforts are needed to maintain the cognitive abilities of the elderly so that their quality of life remains good^1^.

The most common cause is Alzheimer's dementia (AD), characterized by progressive memory decline in the early phase and several cognitive domains and behavioral changes in the late stage^2^. The main neuropathological signs in AD are senile plaque deposition of extracellular Aβ peptides and formation of intracellular neurofibrillary tangles (NFTs) or hyperphosphorylation of tau proteins. In addition, AD is accompanied by chronic neuroinflammation and oxidative stress with synaptic dysfunction and neurodegeneration in various brain areas, including the cortex and hippocampus^3^.

Several previous studies have shown that d-galactose induction to rats causes excessive production of advanced glycation end products (AGEs) and reactive oxygen species (ROS), resulting in cognitive decline. Rats injected with d-galactose showed several features of brain aging, including decreased hippocampal neurogenesis, impaired synaptic plasticity, loss of cholinergic neurons in the basal forebrain, and accumulation of Aβ plaque. Increased accumulation of Aβ senile plaque in the rat brain due to d-galactose injection was associated with an increase in the β-secretase enzyme. Plaque Aβ42, being the most aggressive and neurotoxic form of Aβ plaque, is produced from the breakdown of amyloid precursor protein (APP) by the β-secretase enzyme^4^.

The d-galactose model can be used to study the neurological disorders related to aging, including AD. The d-galactose induction stimulates the effects of aging through the formation of ROS, thereby causing mitochondrial dysfunction, oxidative stress, inflammation, and apoptosis in neurons^5^. The improvements due to d-galactose induction result in an increase in β-secretase enzyme that causes the proteolytic breakdown of APP and increases Aβ42 plaque, which is the most neurotoxic plaque. d-galactose-induced brain aging is dose-dependent, starting from 100 to 500 mg/kg/day for 6–8 weeks^6^. The preelemination study on 20 Wistar rats injected with d-galactose at a dose of 100 mg/kg BW for 8 weeks showed that the mean locomotor activity and spatial memory scores in the intraperitoneal injection group were lower than that in the oral group (p<0.05)^7^.

Neurodegenerative cases including AD started treatment after symptoms began to manifest, even though significant neuron loss had occurred at this stage. AD management with potential therapeutic targets is more effective in preventing dementia in order to increase neurotrophic factors associated with neurotransmission, synaptic plasticity, and elimination of Aβ from the brain^8^. The use of natural ingredients is an interesting option to develop. More and more evidence suggests that flavonoid compounds are effective in preventing the onset and slowing the progression of AD with a direct target of APP metabolism in the amyloid cascade hypothesis. Anthocyanins, a flavonoid compound, have been widely studied to have antioxidant, anti-inflammatory, and antiapoptotic effects^9^. Purple sweet potato extract rich in anthocyanins can modulate protein aggregation and autophagy to improve the disruption of protein homeostasis. These changes correlated with a decrease in apoptosis neurons in the hippocampus and a significant increase in brain-derived neurotrophic factor (BDNF) levels^10^.

Purple sweet potato has a high anthocyanin content and is quite easy to cultivate in tropical climates in Indonesia, including Bali. Further studies are needed to evaluate the benefits of this purple sweet potato extract. To date, no research has been conducted on the effect of purple sweet potato water extract, cultivated in Bali, on dementia rat models with d-galactose induction. Therefore, this research aimed to determine whether purple sweet potato extract has a neuroprotective effect on brain damage caused by oxidative stress, inflammatory processes, and neuronal apoptosis in AD associated with tumor necrosis factor-α (TNF-α) levels, p53 expression, glial fibrillary acidic protein (GFAP) expression, BDNF levels, and spatial working memory.

## METHODS

This study used an experimental animal model with randomized posttest-only control group design.

### Ethical license

This research was approved by the ethics commission of research, Faculty of Medicine, Udayana University (no. 387/UN 14.2.2VII.14/LP/2020).

### Time and place of research

The research was conducted from February to July 2020 at the Integrated Biomedical Laboratory of the Faculty of Medicine, Udayana University, and the Molecular Immunology Laboratory of the Faculty of Veterinary Medicine, Udayana University.

### Research samples

The study samples were taken from affordable populations that met the inclusion and exclusion criteria. Inclusion criteria were male Wistar rats, age 12–14 weeks and weighing 200–300 g. Exclusion criteria were as follows: (a) sick mouse and (b) hyperactive Wistar rat (such as those biting his friend). Exclusion criteria were (a) death of mice during the study period and (b) damage to the sample tissue.

### Research variable

The independent variable is a water extract of purple sweet potato. The dependent variables were TNF-α levels, p53 expression, GFAP expression, BDNF levels, and spatial working memory. The control variables were gender, body weight, age, food, and environmental conditions.

### Research procedure

This study used dementia-induced mice with d-galactose. The analysis of water extract of purple sweet potato was carried out at the Laboratory of Agricultural Product Technology, Udayana University. The TNF-α and BDNF levels were examined using ELISA method. The expressions of p53 and GFAP were detected using immunohistochemical techniques. The spatial working memory was assessed using the Y-maze test.

### Data analysis

- A descriptive analysis was performed on the basic characteristics of the subjects in the two groups (i.e., treatment group and control group), representing mean with standard deviation or median with minimum–maximum as a measure of concentration.
- The data normality test used Shapiro-Wilk test on a sample size of <50. A p>0.05 indicates a normally distributed data.
- Homogeneity test with the Levene test was used to determine data variance.
- Parametric independent t-test was used to find the differences between control and treatment groups on normally and homogeneously distributed data.
- Path analysis was performed to determine the role of purple sweet potato water extract on TNF-α levels, BDNF levels, p53 expression, GFAP expression, and spatial working memory based on its contribution path.
- Test results were assessed using 95 confidence interval (95%CI) and p-value at a significance limit of 0.05.

## RESULTS

### Effect of purple sweet potato water extract on tumor necrosis factor-α levels

The results of the independent t-test analysis in Table 1 show that the water extract of purple sweet potato caused the mean TNF-α level in the treatment group to be significantly lower than the control group after d-galactose induction (p<0.05).

**Table 1:** Differences in tumor necrosis factor-α levels, p53 expression, glial fibrillary acidic protein expression, and brain-derived neurotrophic factor of brain tissue in both groups after observation.

	Levels of brain tissue (pg/mg)					95%CI (min–max)	p-value*
	Groups	n	Mean±SD	Range (min–max)	Mean differences		
TNF-α levels	Control	50	896.77±152.52	665.53–1248.68	161.4	55.79–267.02	0.004†
	Treatment	50	735.36±139.74	436.39–966.57			
p53 expression	Control	50	9.97±2.09	5.29–13.83	3.28	1.88–4.68	<0.001†
	Treatment	50	6.68±1.76	4.36–9.28			
GFAP	Control	50	33.92±4.23	24.70–39.43	8.63	5.79–11.46	<0.001†
	Treatment	50	25.29±13.57	20.09–30.65			
BDNF	Control	50	391.93±140.28	128.02–583.64	106.20	11.46–200.94	0.029†
	Treatment	50	498.13±121.47	321.90–845.79			

95%CI: 95% confidence interval, TNF-α: tumor necrosis factor-α, GFAP: glial fibrillary acidic protein, BDNF: brain-derived neurotrophic factor.

* Student's t-test;

† significant.

### Effect of water extract of purple sweet potato on p53 expression

**Figure 1:**
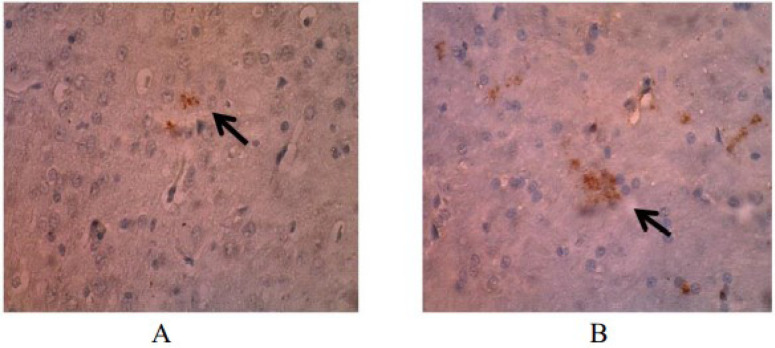
Expression of p53 rat brain on day 70 using immunohistochemical methods (magnification 400×). Astrocyte cells expressing p53 appear brownish (arrow). (A) The treatment group with three neuron cells that express p53. (B) The control group with 12 neuron cells that express p53.

The p53 expression averaged 6.68±1.76% in the treatment group and9.97±2.09% in the control group (Table 1). These results indicated that the water extract of purple sweet potato caused the expression of p53 in the brain tissue of the treatment group to be significantly lower than that in the control group (p<0.001) The expression of p53 in neuron cells examined using immunohistochemistry is presented in Figure 1.

### Effect of water extract of purple sweet potato on glial fibrillary acidic protein expression

**Figure 2:**
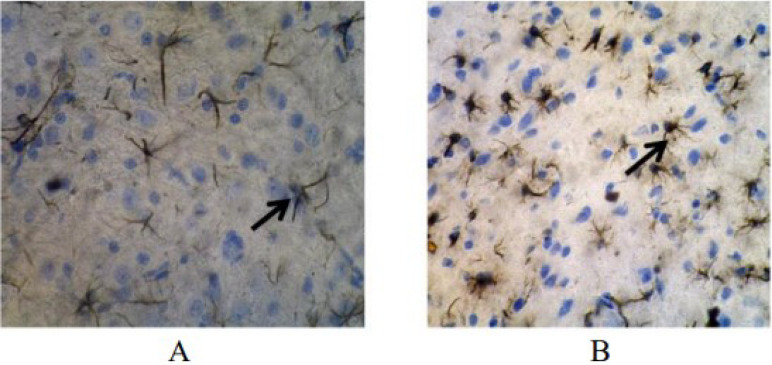
Expression of glial fibrillary acidic proteinin rat brain on day 70 using immunohistochemical method (magnification 400×). Astrocyte cells expressing glial fibrillary acidic proteinappear brownish (arrow). (A) Expression of glial fibrillary acidic proteinin the treatment group with 15 astrocyte cells expressing glial fibrillary acidic protein. (B) The control group with 39 astrocyte cells that express glial fibrillary acidic protein.

The GFAP expression was 25.29±13.57% in the treatment group and 33.92±4.23% in the control group (Table 1). These results indicated that the water extract of purple sweet potato caused the GFAP expression of astrocyte cells in the treatment group to be significantly lower than that in the control group (p<0.001). The GFAP expression of astrocyte cells examined using immunohistochemistry is presented in Figure 2.

### Effect of purple sweet potato water extract on brain-derived neurotrophic factor levels

BDNF levels averaged 498.13±121.47 pg/mg in the treatment group and 391.93±140.28 pg/mg in the control group (Table 1). These results indicated that the water extract of purple sweet potato caused the mean BDNF levels in the treatment group to be significantly higher than that in the control group after d-galactose induction (p=0.029).

### Effect of purple sweet potato water extract on spatial working memory

Based on the results of the independent t-test analysis in Table 2, it can be seen that the baseline spatial working memory before the study (pretest) in the control and treatment groups was not significantly different (p=0.257). After 70 days of observation (posttest), there was a significant difference in spatial working memory in both the groups (p=0.004). The comparative analysis in Table 2 also shows a decrease in spatial memory between the control group before and after the observation from 67.14±12.34 to 59.77±11.87%. These results indicated that injection of d-galactose at a dose of 200 mg/kg BW for 8 weeks caused a decrease in spatial working memory due to the symptoms in dementia mice model. Although purple sweet potato water extract was provided for 70 days, there was an increase in spatial working memory from 61.98±12.93 to 72.01±10.22%.

**Table 2:** Differences of spatial working memory in both the groups before (pretest) and after (posttest) observation.

Memory spatial						95%CI (min–max)	p-value*
Groups		n	Mean±SD	Range (min–max)	Mean differences		
Pretest	Control	50	67.14±12.34	40–81.82	85.16	-3.96 to 14.29	0.257
	Treatment	50	61.98±12.93	37.5–83.33			
Posttest	Control	50	33.92±4.23	24.70–39.43	12.24	4.24 to 20.24	0.004†

95%CI: 95% confidence interval.

* Student's t-test;

† Significant.

### Pathway analysis of the role of purple sweet potato water extract on p53 expression, tumor necrosis factor-α levels, glial fibrillary acidic protein expression, brain-derived neurotrophic factor levels, and spatial working memory

**Figure 3:**
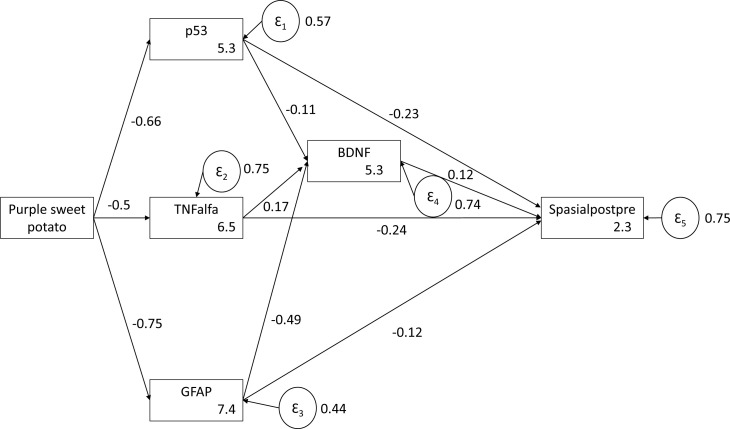
Goodness-of-fit model showing the role of purple sweet potato water extract on p53 expression, tumor necrosis factor-α levels, glial fibrillary acidic protein expression, brain-derived neurotrophic factor levels, and spatial working memory based on path analysis.

The role of purple sweet potato water extract on p53 expression, TNF-α levels, GFAP expression, BDNF levels, and spatial working memory based on the contribution pathway for each variable is presented simultaneously in the path analysis shown in Figure 3. Evaluation of model suitability (goodness of fit) describes how well or fit a series of variable observations to the model. Based on the goodness-of-fit index, this model is declared fit with root mean square error of approximation (RMSEA)<0.08.

This model explains the effect of purple sweet potato water extract on memory enhancement. The results of this pathway analysis show that purple sweet potato extract suppresses oxidative stress and inflammation, as indicated by its negative effect on the three biomarkers, namely, p53 expression, TNF-α levels, and GFAP expression. The suppression of oxidative stress and inflammatory biomarkers has a direct or an indirect effect on increasing spatial working memory by increasing BDNF levels. The effect of one variable on other variables in this study is shown in Table 3.

**Table 3:** Direct effect, indirect effect, and the total effect of one variable on other variable.

	Effect					
	Direct		Indirect		Total	
	β	p-value*	β	p-value*	β	p-value*
Purple potato on p53	-0.659	<0.001†	–	–	-0.659	<0.001†
Purple potato on TNF-α	-0.495	<0.001†	–	–	-0.495	<0.001†
Purple potato on GFAP	-0.754	<0.001†	–	–	-0.754	<0.001†
Purple potato on BDNF	–	–	0.355	0.013†	0.355	0.013†
Purple potato on memory	–	–	0.400	0.004†	0.400	0.004†
P53 on BDNF	-0.113	0.522	–	–	-0.113	0.522
P53 on memory	-0.232	0.188	-0.013	0.645	-0.246	0.171
TNF-α on BDNF	0.173	0.295	–	–	0.173	0.295
TNF-α on memory	-0.235	0.158	0.020	0.573	-0.215	0.202
GFAP on memory	-0.488	0.003†	–	–	-0.488	0.003†
GFAP on BDNF	-0.117	0.556	-0.058	0.516	-0.175	0.334
BDNF on memory	0.119	0.501	–	–	0.119	0.501

TNF-α: tumor necrosis factor-α, GFAP: glial fibrillary acidic protein, BDNF: brain-derived neurotrophic factor.

* Student's t-test;

† significant; β=standardized coefficient.

## DISCUSSION

The purple sweet potato water extract affected memory enhancement through its direct effect on suppression of oxidative stress and inflammatory pathways, such as p53 expression, TNF-α levels, and GFAP expression. The highly significant direct effect (p<0.05) was through the suppression of the GFAP activation pathway by 75.1%. Overall, from the total effect, it appears that purple sweet potato water extract has a significant indirect effect on improving memory. The results of this study indicate that purple sweet potato extract has the ability to suppress oxidative stress and inflammation and therefore inhibits apoptosis and triggers neurogenesis, thereby causing improvement in spatial memory.

The increase in memory is not solely through the BDNF pathway. There is a direct effect due to the suppression of the inflammatory process itself, and if inflammation is suppressed, there is a direct effect on memory improvement. Although there is an increasing understanding of the pathogenesis of AD, accurate evidence on the mechanism of the disease is still lacking. The focus therapy so far has largely targeted the excitotoxicity and cholinergic hypotheses, but recently there is an increasing evidence of the importance of non-neuronal cells such as astrocytes. This opens new research avenues to better understand disease pathology with the molecular target of astrocytes for drug development. Astrocytes have a role in protecting neurons in physiological conditions, and astrocyte dysfunction triggers neuronal degeneration, which interferes with synapse delivery and causes cognitive impairment that occurs in AD. Astrocytes become reactive due to the deposition of amyloid plaque, resulting in decreased glutamate uptake due to reduced transporter expression, changes in energy metabolism, disruption of K and Ca ion homeostasis, and the release of cytokines and inflammatory mediators^11^.

Reactive astrocytes show changes in astrocyte function characterized by an increased expression of a number of astrocyte structural proteins, such as GFAP and vimentin. Morphological changes in reactive astrocytes such as hypertrophy and proliferation of processes are important in the formation of astrocyte scar around the lesion tissue. Continuous astrocyte reactivity is triggered by a positive feedback cycle between microglia and astrocytes in a prolonged brain disorder that disrupts neuronal function and leads to chronic neuroinflammation. The etiology of neurodegenerative diseases is largely unknown; however, there are a number of contributing factors including oxidative stress, calcium excitotoxicity, neuroinflammation, and disruption of protein homeostasis, leading to neuronal apoptosis in the brain. Anthocyanin polyphenol compounds present in purple sweet potato extract are able to modulate many of the pathways underlying neurodegenerative diseases so that their development is promising as multitarget drug ligands (MTDLs) for the prevention and treatment of neurodegenerative diseases. The neuroprotective effect of anthocyanins is inseparable from the evidence showing its ability to cross the blood–brain barrier^10^.

**Figure 4:**
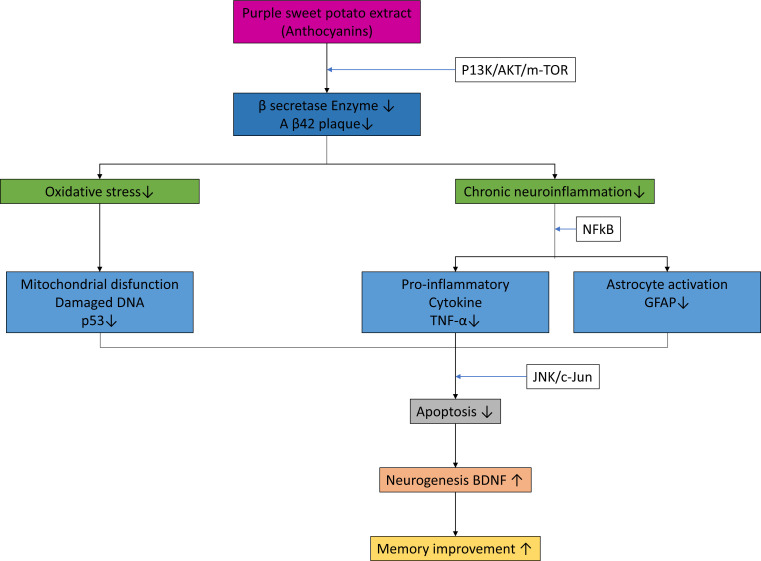
Schematic illustration of the mechanism of anthocyanin gives the effect of neuroprotective through decreased expression of p53, the levels of tumor necrosis factor-α, the expression of glial fibrillary acidic protein and increased brain-derived neurotrophic factor and spatial working memory.

Purple sweet potato water extract with the main content of anthocyanins has been shown to have a direct effect in suppressing oxidative stress and inflammation, thereby triggering apoptosis and causing neuroprotective effects, such as an increase in neurogenesis and improvement of spatial working memory. These results support previous research by Wurzelmann et al.^12^ who demonstrated the neuroprotective role of anthocyanins on the mechanisms of oxidative stress and inflammation in dementia mice model. Based on the results of the research and discussion, an illustration scheme was made. The anthocyanin mechanism provides a neuroprotective effect by decreasing p53 expression, TNF-α levels, and GFAP expression and increasing BDNF and spatial working memory (Figure 4).

Anthocyanins have been shown to act as antioxidants that directly bind ROS, and reactive nitrogen species (RNS) were assessed directly by their high oxygen radical absorption capacity (ORAC) and indirectly by increasing intrinsic antioxidant defenses through increasing levels and activity of antioxidant enzymes such as catalase, SOD, and GSH through activation of the transcription factor Nrf2. In this study, it was proven that anthocyanins have antioxidant activity by suppressing DNA damage and apoptosis, which is judged by low p53 expression. Normal p53 bound with the mouse double minute 2 (MDM2) triggers ubiquitination so that p53 levels are low and do not cause apoptotic effects. If there is DNA damage, p53 functions to prevent replication in damaged cells by stopping the cell cycle in the G1 phase (interphase) so that the cells have time to repair themselves. However, the failure of repairing cells or extensive damage will trigger apoptosis. Apoptosis is triggered by p53 through the intrinsic pathway when DNA damage is triggered by oxidative stress, ATM/ATR, and CHK2 proteins are released so that p53 does not bind to MDM2 and p53 accumulates. Increased p53 will inhibit the activity of antiapoptotic protein (Bcl-2) and stimulate pro-apoptotic BH3-only proteins such as Bim, PUMA, NOXA to activate Bax and Bak proteins to form oligomers on the mitochondrial surface of a channel called MOMP (mitochondrial outer membrane permeability), which triggers the apoptotic cascade. This channel causes the release of cytochrome c to the cytoplasm, which combines with APAF-1 and caspase 9 to form the apoptosis. The apoptosis breaks down procaspase 3 into active caspase 3, which stimulates apoptosis. This study also proved the anti-inflammatory ability of anthocyanins based on TNF-α levels and GFAP expression. The inflammatory process in dementia is triggered by the presence of Aβ protein that forms Aβ plaque. Toxic plaque triggers oxidative stress on the astrocytes and microglia around the plaque so that the microglia is activated and the astrocytes become reactive. Plaque Aβ induces activation of the microglia via TLR and RAGE signals, resulting in activation of Erk1/2, Akt, p38, and MAPK in order to activate transcription factor NFκB and triggering the release of pro-inflammatory mediators such as TNF-α, interleukin (IL)-1B, and IL-6. Inflammatory mediators in neurons cause increased amyloid production. This communication between neurons and glia further strengthens neurotoxicity, especially in the cholinergic neurons in the basal forebrain, which are the targets of AD. As a ligand, TNF-α will bind to the receptor. TNFR-1 triggers trimerization to bind to TRADD and FADD adapter proteins to form DISC so that procaspase 8 proteolysis becomes active caspase 8, which triggers apoptosis directly by activating caspases 3, 6, and 7. Astrocytes have the main function of protecting neurons and repairing tissue after injury/stress by forming scar glia (astrogliosis), which serves to resist the spread of inflammation and repair damage to the blood–brain barrier. Astrocytes near the amyloid plaque become reactive after oxidative stress. Reactive astrocytes are characterized by hypertrophy in the astrocyte process, leading to the release of the structural protein GFAP. In this study, neuroprotective effects of anthocyanin were obtained indirectly through the BDNF pathway. BDNF is a neurotrophic factor that plays an important role in synaptic plasticity and neuronal cell survival. BDNF binds to the tyrosine kinase B (TrkB) receptor to activate the PI3K/Akt pro-survival pathway. The activated PI3K produces phosphatidylinositol that will react with phosphatidylinositol-dependent kinase (PDK) and serine/threonine and form phosphorylated Akt. The Akt will activate the antiapoptotic gene and inhibit the transcription factors that trigger apoptosis and activate mammalian target of rapamycin (mTOR) which will activate and phosphorylate apoptosis genes, such as *MCL-1*. It also phosphorylates MDM2, thereby inhibiting p53 activation and phosphorylating CREB on Ser 133 residues to recruit CREB-binding protein that regulates antiapoptotic Bcl-2 regulation and will inhibit the Forkhead box O3 (FOXO3), which activates Bim and NOXA. However, in this study, it was found that although the neuroprotective effect of anthocyanins was not solely through the BDNF pathway, there was a direct effect due to the suppression of the inflammatory process itself, and if inflammation was suppressed, there was a direct effect on memory improvement. This is probably due to anthocyanins exhibiting a direct neuroprotective effect by preventing protein aggregation and stimulating autophagy, thereby suppressing inflammation. Protein aggregation plays an important role in neuronal death by inhibiting the oligomerization of amyloid proteins into toxic fibrils and plaques. Anthocyanin cyanidin-3-*O*-pure glucopyranoside have been shown to directly inhibit the oligomerization of Aβ peptides^13^. Likewise, malvidin and cyanidin-3-*O*-glucopyranoside were reported to have the potential to inhibit the oligomerization of Aβ into toxic fibrils^14^. This finding was further corroborated by other studies showing that pure anthocyanins and anthocyanidins prevent Aβ oligomerization directly in neuron cells, thereby preventing the formation of protein aggregates in AD^15^. Although the mechanism by which anthocyanins inhibit aggregate formation is not currently known, the ability of these compounds to inhibit the formation of toxic oligomers holds promise for therapeutic success. Recent studies also report the ability of anthocyanin-rich extracts to modulate autophagy to clear toxic protein aggregates from the intracellular space to prevent neuronal death. Anthocyanins significantly increase autophagosomes turnover and increase the activation of mTOR, one of the regulators of the autophagy pathway^16^. Similarly, a more recent study showed that extracts from purple sweet potato significantly increased autophagy markers in the hippocampus of rats fed a high-fat diet and showed that this process was dependent on protein kinase (AMPK) activation^17^. This change correlates with a decrease in neuronal apoptosis in the hippocampus and a significant increase in BDNF^16^. Overall, these results suggest that anthocyanins and anthocyanin-rich extracts can modulate processes such as protein aggregation and autophagy to correct the disruption of protein homeostasis in AD, although further data are needed to confirm this hypothesis.

The results of this study strengthen the theory of inflammation and oxidative stress as factors that play an important role in the pathogenesis of dementia and have succeeded in proving the neuroprotective effect of purple sweet potato water extract. This is shown from the invention: (1) TNF-α levels in mice with induction of d-galactose given purple sweet potato water extract were lower than those not given purple sweet potato water extract. (2) The expression of p53 in mice with induction of d-galactose given purple sweet potato water extract was lower than those that were not given purple sweet potato extract. (3) The expression of GFAP in mice with d-galactose induction given purple sweet potato water extract was lower than those not given purple sweet potato water extract. (4) BDNF levels in mice with induction of d-galactose given purple sweet potato water extract were higher than those not given purple sweet potato water extract. (5) Spatial working memory in mice with induction of d-galactose given purple sweet potato water extract increased compared to those not given purple sweet potato water extract.
